# Impact of Patient Selection on Performance of an Early Rule-Out Pathway for Myocardial Infarction: From Research to the Real World

**DOI:** 10.1161/CIRCULATIONAHA.122.062419

**Published:** 2023-01-31

**Authors:** Anda Bularga, Kuan Ken Lee, Anoop S.V. Shah, Atul Anand, Andrew R. Chapman, Chris Tuck, David E. Newby, Sarah Jenks, Nicholas L. Mills, Dorien M. Kimenai

**Affiliations:** 1 BHF Centre for Cardiovascular Science (A.B., K.K.L., A.A., A.R.C., C.T., D.E.N., N.L.M., D.M.K.), University of Edinburgh, United Kingdom.; 2 Usher Institute (A.A., N.L.M.), University of Edinburgh, United Kingdom.; 3 Department of Non-communicable Disease, London School of Hygiene and Tropical Medicine, United Kingdom (A.S.V.S.).; 4 Department Clinical Biochemistry, Royal Infirmary of Edinburgh, United Kingdom (S.J.).

**Keywords:** acute coronary syndrome, myocardial infarction, troponin I

Early rule-out pathways for myocardial infarction using high-sensitivity cardiac troponin are now recommended by international clinical guidelines.^1^ Although developed in selected patients participating in research studies, these pathways are applied widely in clinical practice where the diagnostic performance and effectiveness may differ substantially. We previously demonstrated that the selection of patients for high-sensitivity cardiac troponin testing influences the apparent prevalence of myocardial infarction.^2^ Here, we evaluated the impact of patient selection on the performance of the High-STEACS trial (High-Sensitivity Troponin in the Evaluation of Patients with Acute Coronary Syndrome) early rule-out pathway for myocardial infarction^3^ in unselected consecutive patients in clinical practice and in selected patients consenting to participate in a research study.

Both cohorts comprised patients with suspected acute coronary syndrome presenting to emergency departments across 3 acute-care hospitals in Scotland. An electronic form embedded in practice was used to record prospectively the indication for cardiac troponin testing, symptoms, and timing of symptom onset. In the unselected cohort, data were collected in consecutive patients as part of a service evaluation in which the usual care clinician measured cardiac troponin for suspected acute coronary syndrome between June 7, 2021, and September 8, 2021. Data collection and record linkage were performed with approval from the Caldicott Guardian and the ethics committee. In the selected cohort, patients with suspected acute coronary syndrome were enrolled by researchers between June 1, 2013, and March 31, 2017, as described previously.^4^ In this cohort, patients were approached between 8:00 am and 8:00 pm by research staff, and written informed consent was obtained. This study was registered (https://www.clinicaltrials.gov; Unique identifier: NCT01852123) and approved by the ethics committee. Patients presenting with ST-segment–elevation myocardial infarction were excluded. These studies make use of multiple routine electronic health care data sources that are linked, deidentified, and held in our national safe haven that is accessible by approved individuals who have undertaken the necessary governance training.

In both cohorts, presentation and serial cardiac troponin concentrations were measured using the ARCHITECT_*STAT*_ high-sensitive troponin I assay (Abbott Laboratories) and sex-specific 99th percentile diagnostic thresholds applied (16 ng/L for women, 34 ng/L for men). We assessed the performance of the High-STEACS early rule-out pathway for an adjudicated diagnosis of myocardial infarction (type 1, 4b, or 4c) during the index hospital admission. Patients presenting within 2 hours of symptom onset (early presenters) underwent serial testing. Having access to the same clinical information in both cohorts, adjudication was performed independently by 2 cardiologists with consensus from a third where required, according to the Fourth Universal Definition of Myocardial Infarction.^5^

The unselected and selected patient cohorts comprised 1242 (median age, 60 [interquartile range, 47–75] years, 46% women) and 1695 (median age, 61 [interquartile range, 52–73] years, 40% women) patients, respectively. Chest pain was the primary symptom in 91% (1128/1242) and 84% (1426/1695) of patients in the unselected and selected cohorts, respectively. The median time from symptom onset was 4 (interquartile range, 3–6) hours in the unselected cohort and 3 (interquartile range, 2–9) hours in the selected cohort. At presentation, cardiac troponin concentrations were elevated above the sex-specific 99th percentile in 14% (177/1242) and 15% (247/1695) of unselected and selected patients. On serial testing, concentrations were elevated in 17% (204/1242) and 19% (330/1695), respectively, but the proportion with a final diagnosis of myocardial infarction differed at 6% (74/1242) and 14% (232/1695), respectively.

After the initial cardiac troponin measurement, the High-STEACS pathway identified 54% (676/1242) of unselected patients and 36% (613/1695) of selected patients as low risk. Overall, more patients had myocardial infarction ruled out by the pathway in the unselected cohort than in the selected cohort (77% [958/1242] versus 66% [1119/1695]; *P*<0.001), with a similar negative predictive value (99.9% [95% CI, 99.8%–100%] versus 99.8% [95% CI, 99.4%–99.9%]) and sensitivity (99.3% [95% CI, 97.4%–100%] versus 98.9% [95% CI, 97.6%–99.9%]; Figure). In the selected cohort, more patients had intermediate troponin concentrations (36% [612/1695] versus 29% [353/1242]) requiring serial testing and more patients were ruled in (34% [576/1695] versus 23% [284/1242]; *P*<0.001 for both). The positive predictive value for myocardial infarction was lower in the unselected patient cohort (26.1% [95% CI, 21.2%–31.4%] versus 39.9% [95% CI, 36.0%–44.0%]; *P*<0.001, Figure) for a similar specificity (82.0% [95% CI, 79.8%–84.2%] versus 76.3% [95% CI, 74.1%–78.5%]).

**Figure.**
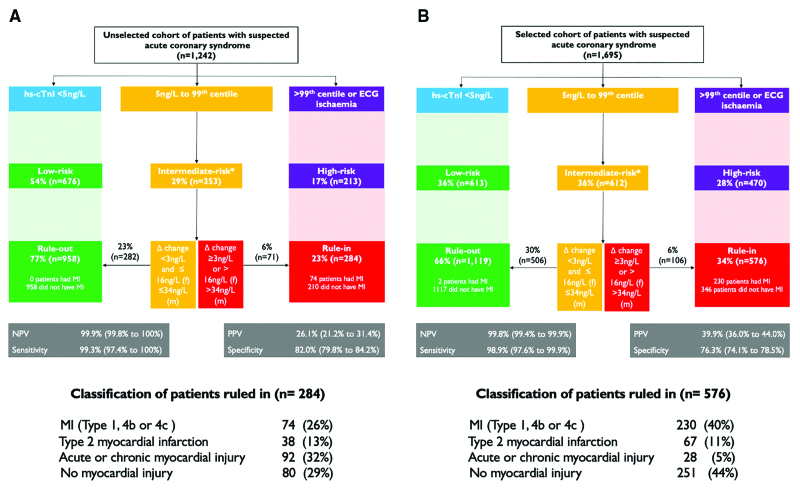
**Performance of the High-STEACS early rule-out pathway for myocardial infarction in an unselected patient cohort from clinical practice (A) and in a selected patient cohort participating in a research study (B)** All patients were adjudicated according to the Fourth Universal Definition of Myocardial Infarction, and the classification of those ruled in by the pathway is given at the bottom the Figure. The pathway recommends that patients presenting within 2 hours of symptom onset have serial measurements. *Serial high-sensitivity cardiac troponin I (hs-cTNI) concentrations were not available in 130 patients in the unselected patient cohort (**A**) and in 17 patients in the selected patient cohort (**B**). High-STEACS indicates High-Sensitivity Troponin in the Evaluation of patients with Acute Coronary Syndrome; MI, type 1, 4b, or 4c myocardial infarction; NPV, negative predictive value; and PPV, positive predictive value.

Our findings highlight that the performance of early rule-out pathways for acute myocardial infarction that have been developed in selected patient cohorts are likely to differ substantially when applied in clinical practice because of the lower prevalence of myocardial infarction. As a consequence, fewer patients are ruled in, and the positive predictive value of cardiac troponin for myocardial infarction is lower in practice. However, more patients are ruled out in clinical practice, resulting in greater efficacy of early rule-out pathways without compromising safety.

## Article Information

### Acknowledgments

Drs Bularga, Mills, and Kimenai conceived the study and its design. Drs Bularga and Kimenai had access to the data and performed the analysis. Drs Bularga, Mills, and Kimenai interpreted the data and drafted the manuscript. All authors revised the manuscript critically for important intellectual content and provided their final approval of the version to be published. All authors are accountable for the work. The authors thank researchers from the Emergency Medicine Research Group Edinburgh for their support during the conduct of High-STEACS trial. The authors thank the Clinical Biochemistry Department staff at Royal Infirmary of Edinburgh and the NRS Bioresource team for their support with sample collection and analysis.

### Sources of Funding

The British Heart Foundation (SP/12/10/29922) funded the High-STEACS trial with support from a Research Excellence Award (RE/18/5/34216). Dr Bularga is supported by a Clinical Research Training Fellowship (MR/V007254/1) from the Medical Research Council. Dr Mills is supported by a Chair Award (CH/F/21/90010), a Programme Grant (RG/20/10/34966), and a Research Excellence Award (RE/18/5/34216) from the British Heart Foundation. Dr Kimenai is supported by a grant from Health Data Research UK, which receives its funding from HDR UK Ltd (HDR-5012) funded by the UK Medical Research Council, Engineering and Physical Sciences Research Council, Economic and Social Research Council, Department of Health and Social Care (England), Chief Scientist Office of the Scottish Government Health and Social Care Directorates, Health and Social Care Research and Development Division (Welsh Government), Public Health Agency (Northern Ireland), British Heart Foundation and the Wellcome Trust. Abbott Laboratories provided cardiac troponin assay reagents, calibrators, and controls without charge.

### Disclosures

Dr Shah’s institution has received honoraria from Abbott Diagnostics. Dr Mills reports research grants awarded to the University of Edinburgh from Abbott Diagnostics and Siemens Healthineers outside the submitted work, and honoraria from Abbott Diagnostics, Siemens Healthineers, Roche Diagnostics and LumiraDx. All other authors have no interest to declare.
